# Indocyanine Green and Technetium-99m: CT-Guided Marking of Lung Nodules Before Robotic-Assisted Lung Resection

**DOI:** 10.3390/diagnostics16213623

**Published:** 2026-11-02

**Authors:** Francesco Ferrante, Sara Ricciardi, Guido Ventroni, Alessandro Semprebene, Enrico Pampana, Luca Bertaccini, Enrico Melis, Maria Cristina Macciomei, Andrea Tornese, Giuseppe Cardillo

**Affiliations:** 1 Thoracic Surgery Unit, San Camillo-Forlanini Hospital, 00152 Rome, Italy; francescoferrante1995@gmail.com (F.F.); sricciardi2@scamilloforlanini.rm.it (S.R.); gcardillo@scamilloforlanini.rm.it (G.C.); 2 Nuclear Medicine Unit, San Camillo-Forlanini Hospital, 00152 Rome, Italy; 3 Interventional Radiology Unit, San Camillo-Forlanini Hospital, 00152 Rome, Italy; 4 Pathology Unit, San Camillo-Forlanini Hospital, 00152 Rome, Italy; mmacciomei@scamilloforlanini.rm.it (M.C.M.);

**Keywords:** RATS lung resection, lung nodule localization, nodule marking, sublobar lung resection

## Abstract

**Background/Objectives:** For stage I non-small cell lung cancer, surgery remains the cornerstone of treatment and the use of minimally invasive techniques is desirable. The lack of tactile feedback in RATS is a well-known drawback of this approach, so precise identification of the disease is crucial for performing lung resection. **Methods:** We present our retrospective case history, accounting for 83 patients, which were divided into two groups based on marking technique: 99^m^Tc-MAA (99^m^Technetium Albumin Macroaggregates) only (Tech group) (22 cases, 26.5%) and 99^m^Tc-MAA + Indocyanine Green (Tech + ICG group) (61 cases, 73.49%). **Results:** In the Tech group, there were 11 males (50%) ad 11 females (50%), the median nodule diameter was 21.5 mm (IQR 16), and the median distance of the nodule from the visceral pleura was 18 mm (IQR 21). Eighteen out of 22 nodules (81.82%) were subsolid. Three conversions were reported, all due to failure to identify the nodule (13.63%). The mean operative time was 111.6 ± 45.6 min. The histopathological examination resulted in 13 lung adenocarcinomas, one squamous cell carcinoma, two minimally invasive lung adenocarcinomas, two lung adenocarcinomas in situ, three metastases from another tumor, and one benign nodule. In the Tech + ICG group, there were 27 males (44.26%) and 34 females (55.73%), the median nodule diameter was 18 mm (IQR 14), and the median distance of the nodule from the visceral pleura was 24 mm (IQR 15). Forty-six out of 61 nodules were subsolid (75.4%). Four conversions were reported, due to failure in nodule identification (6.55%). The operative time was 105.6 ± 36.8 min. The histopathological examination resulted in 12 lung adenocarcinomas, seven squamous cell carcinomas, nine minimally invasive lung adenocarcinomas, 12 lung adenocarcinomas in situ, one typical carcinoid, seven atypical adenomatous hyperplasias, seven metastases from another tumor, and six benign nodules. The two groups are homogeneous in terms of median nodule diameter (*p* = 0.676), the median distance from visceral pleura (*p* = 0.114), SUV max (*p* = 0.894), operative time (*p* = 0.512), and nodule characteristics (solid vs. subsolid) (*p* = 0.724). The conversion rate was lower in the Tech + ICG group (6.55% vs. 13.63%), but the difference was not statistically significant (*p* = 0.375). **Conclusions:** Both techniques appear to be safe and reproducible. Further standardized studies are needed to validate and confirm the results obtained from our case series.

## 1. Introduction

Increasing use of computed tomography (CT) scans has led to the detection of small-size lung nodules, including solid and subsolid ones [1]. For stage I non-small cell lung cancer (NSCLC), surgery remains the cornerstone of treatment and the use of minimally invasive techniques is desirable. Concurrently, the surgical management of early-stage NSCLC has undergone a paradigm shift after the results of the JCOG0802/WJOG4607L and CALGB 140503 trials [2,3]. Sublobar resection, including lung segmentectomy and wedge resection, has emerged as a valuable option for <2 cm peripheral lung nodules. In addition, other efforts have focused on the management of part-solid lesions and pure ground-glass opacities (GGOs), and the role of lymphadenectomy in these lesions [4].

However, the transition toward parenchymal-sparing surgery presents significant technical issues, particularly in the context of Robotic-Assisted Thoracic Surgery (RATS). RATS provides superior visualization and greater maneuverability compared to conventional Video-Assisted Thoracic Surgery (VATS). However, the lack of tactile feedback in RATS is a well-known drawback of this approach, so the precise identification of the disease is crucial for performing lung resection, especially in cases where there is no preoperative histopathological diagnosis. This absence of tactile sensation makes the intraoperative identification of small, deep-located, or non-solid nodules challenging. Furthermore, unlike VATS, in RATS the movement of the instruments cannot be used to palpate the visceral pleura in order to detect changes in density consistent with the presence of a nodule. Without precise localization, there is an increased risk of inadequate surgical margins, prolonged operative times, or the need for an unwanted conversion to thoracotomy.

The widespread implementation of lung cancer screening programs has led to a substantial increase in the detection of pulmonary nodules. While this has contributed to earlier diagnosis and improved survival in selected populations, it has simultaneously provided a growing cohort of lung nodules lacking a certain histological diagnosis [5]. The majority of these lesions are small, peripheral, and often non-palpable, posing significant challenges for intraoperative localization and diagnostic assessment. Consequently, the ability to accurately identify and localize pulmonary nodules has become a critical step in the diagnostic and therapeutic pathway.

Moreover, the increasing prevalence of subsolid nodules and ground-glass opacities, which are lesions often associated with early-stage adenocarcinoma [6], highlights the importance of accurate intraoperative identification. These lesions are particularly challenging due to their subtle radiological appearance and lack of distinct anatomical limits.

To overcome these limitations, several preoperative localization techniques have been developed, ranging from the use of metallic hooks and coils to the injection of liquid dyes [7,8]. Among these, localization using 99^m^Tc-MAA has proven to be a reliable method [9]. The emission of gamma radiation allows for trans-parenchymal detection using a gamma probe, which displays the recorded radioactivity using a numerical scale and an audible signal.

In the last decade, near-infrared (NIR) fluorescence imaging using Indocyanine Green (ICG) has gained consensus [10,11]. In the literature, ICG has been injected directly on the segment of interest by mean of bronchoscopy. When injected peritumorally under CT guidance, ICG provides a clearly defined fluorescent mark on the pleural surface because of lung lymphatic drainage.

Despite the theoretical benefits of combining these tracers, there is a need for clinical data comparing the efficacy and safety of the mixed-technique approach versus fluorescence or Tc-99m alone. This study aims to evaluate and compare the surgical outcomes, localization success rates, and complication profiles of patients undergoing pulmonary nodule localization with 99^m^Tc-MAA alone versus a combined 99^m^Tc-MAA and ICG approach in the setting of RATS lung resection. These two groups were formed following a change in protocol at our institution, which resulted from our previous experience with VATS surgery and the start of our robotic surgery activity.

## 2. Materials and Methods

We conducted a study to evaluate the efficacy, safety, and reproducibility of preoperative pulmonary nodule localization using two different markers: Tc-99m radiocolloid alone (Tech Group, 22 patients) and the combination of Tc-99m-MAA and Indocyanine Green (Tech + ICG Group, 61 patients). This is a single-center retrospective study that enrolled 83 patients from January 2019 to February 2026. Each patient was scheduled to undergo RATS lung resection. For every patient, demographic data were recorded (age at surgery, sex, Body Mass Index (BMI), smoker status, and pack years). The study was conducted in accordance with the Declaration of Helsinki and approved by the Institutional Review Board of the Cardio-Thoraco-Vascular Department of Hospital San Camillo-Forlanini (protocol number 002326, approved on 13 March 2026). All patients signed an informed consent form for both the nodule localization procedure and the surgical intervention.

None of the patients had a preoperative histological diagnosis, so in no case was an anatomic lung resection preoperatively scheduled. In addition to a whole-body CT scan for staging, all patients also underwent a PET-CT (positron emission tomography) scan with ^18^FDG (18-fluorodeoxyglucose); nodule diameter, depth from visceral pleural, lobe of belonging, and SUV max (maximum standard uptake value) were recorded for each patient. Nodule radiological characteristics were classified as pure GGO nodule, solid nodule, part-solid nodule with CTR < 0.5 (consolidation to tumor ratio), and part-solid nodule with CTR > 0.5, according to Fleischner 2017 guidelines [1]. The recorded surgical data included operative time, conversion to open surgery, procedure complications, surgical complications, length of stay, and failure of marking technique.

The marking procedure was performed the day before the intervention (about 20 h before the surgery) because, based on our previous experience, ICG distributed more effectively and with less spread on the pleural surface when performed at this time. This application timing results in a clearer indication of the nodule’s location compared to marking on the morning of the intervention; in this latter case, there is greater spread and less precise distribution on the visceral pleura.

In both groups, the procedure was performed under CT guidance by two interventional radiologists experienced in lung nodule biopsy and marking (4–5 procedures per week for over ten years). In the Tech Group, 20–25 MBq of 99^m^Tc-MAA (Pulmocis, Curium Pharma/Curium Italy S.R.L., Milan, Italy, Global Medical Solutions NZ, 2/110 Mays Road, Onehunga, Auckland 1061, New Zealand) was injected. In the Tech + ICG Group, 25 mg of ICG powder (Diagnostic Green Limited, Athlone Business and Technology Park, Garrycastle, Athlone, Co. Westmeath, N37 F786, Ireland), at a concentration of 5 mg/mL, was dissolved in 5 mL of saline solution, and 0.3 mL of this solution was then injected together with 99^m^Tc-MAA. At the start of the experience, the radiologist injected the solution directly into the nodule in some cases, based on the accessibility and the depth of the nodule. In such cases, the pathologist reported the presence of a neutrophilic inflammatory infiltrate into and nearby the lesion. For this reason, direct injection into the nodule was deliberately avoided, and injection near the lesion was preferred. This change did not affect the precision of nodule marking. At least 5 h after the procedure, each patient underwent a chest X-ray to check for pneumothorax and/or hemothorax, even though the presence of pneumothorax is routinely assessed by CT scan immediately after the procedure.

Our standard approach for lung resection and lymphadenectomy procedures is a fully robotic four-port approach with carbon dioxide insufflation; three 8 mm incisions and one 12 mm anterior incision were used for surgical specimen extraction. The robotic platform that we used is the Da Vinci Xi, and the method used to detect fluorescence is the Firefly mode integrated in the platform.

During surgery, the Tech group relied only on a handheld gamma probe to detect radioactivity. In the Tech + ICG group, localization was achieved at first through NIR fluorescence imaging, allowing for real-time visual identification of the region on the lung surface undergoing resection. Then, localization was confirmed by inserting the gamma probe in the chest, via the 12 mm port access, or once the histological specimen was removed.

### Statistics

The data are presented as the median ± interquartile range (IQR) for non-normally distributed data or as the mean ± standard deviation (SD) for normally distributed data. Statistical comparisons were performed using the Student’s *t*-test or Mann–Whitney U test for continuous variables, and the Chi-square or Fisher’s exact test for categorical variables. A *p*-value < 0.05 was considered statistically significant. The data was processed using Microsoft Excel v16.108.3 and IBM SPSS Statistics v30.

## 3. Results

In the Tech group, there were 11 males (50%) and 11 females (50%), the median age was 50.5 years (IQR 28.25), and the median BMI was 28.7 kg/m^2^ (IQR 7.2). Six patients were current smokers and 11 were former smokers, with a mean pack years number of 34.88 (SD ± 19.18). Three nodules were located in the right lower lobe, four in the left upper lobe, six in the middle lobe, five in the right upper lobe, and four in left lower lobe. The median nodule diameter was 21.5 mm (IQR 16), the median distance of the nodule from the visceral pleura was 18 mm (IQR 21), and the mean SUV max was 5.54 (SD ± 3.22). Eighteen out of 22 nodules (81.82%) were subsolid: five pure GGO nodules, seven nodules with CTR < 0.5, and six nodules with CTR > 0.5. Four out of 22 nodules were solid (18.18%). After the marking procedures, four complications (18.18) were observed: three minor pneumothoraxes were reported and one pulmonary hematoma, which were all treated conservatively. Three conversions were reported, all due to failure to identify the nodule (13.63%). The mean operative time was 111.6 min (SD ± 45.6). The length of stay was 6 days (SD ± 2.8). The histopathological examination resulted in 13 lung adenocarcinomas, one squamous cell carcinoma, two minimally invasive lung adenocarcinoma, two lung adenocarcinoma in situ, three metastases from another tumor, and one benign nodule.

In the Tech + ICG group, there were 27 males (44.26%) and 34 females (55.73%), the median age was 44 years (IQR 31), and the median BMI was 27 kg/m^2^ (IQR 8.9). Twenty-four patients were current smokers and 20 were former smokers, with a mean pack years number of 33.52 (SD ± 16.39); 17 patients were never smokers. Sixteen nodules were located in the right lower lobe, 18 in the left upper lobe, 11 in the middle lobe, nine in the right upper lobe, and seven in the left lower lobe. The median nodule diameter was 18 mm (IQR 14), the median distance of the nodule from the visceral pleura was 24 mm (IQR 15), and the mean SUV max was 5.69 (SD ± 3.23). Forty-six out of 61 nodules were subsolid (75.4%): 19 pure GGO nodules, 17 nodules with CTR < 0.5, and 10 nodules with CTR > 0.5. Fifteen out of 61 nodules were solid (24.6%). After the marking procedures, three minor pneumothoraxes (4.91%) were reported, all of which were treated conservatively. Four conversions were reported, due to failure in nodule identification (6.55%). Mean operative time was 105.6 (SD ± 36.8). Length of stay was 5.5 days (SD ± 2.9). The histopathological examination resulted in 12 lung adenocarcinomas, seven squamous cell carcinomas, nine minimally invasive lung adenocarcinomas, 12 lung adenocarcinomas in situ, one typical carcinoid, seven atypical adenomatous hyperplasia, seven metastases from other tumors, and six benign nodules.

The two groups are homogeneous in terms of median nodule diameter (*p* = 0.676), median distance from the visceral pleura (*p* = 0.114), SUV max (*p* = 0.894), operative time (*p* = 0.512), and nodule characteristics (solid vs. subsolid) (*p* = 0.724). No statistically significant difference was observed in the failure of techniques between the two groups: Tech 13.63% vs. Tech + ICG 6.55%, (*p* = 0.375). The table below summarizes the patients’ characteristics (Table 1).

## 4. Discussion

The accurate localization of small pulmonary nodules remains a challenge in thoracic surgery, particularly with the increasing use of minimally invasive techniques and the lack of tactile feedback that characterizes RATS.

The literature reports several experiences regarding lung nodule marking. Ichinose et al. [8] classified lung nodule localization in three groups: (1) methods based on ultrasonography or pressure sensors; (2) percutaneous insertion of hook wires, dyes, fluorescent agents, and radioactive agents; and (3) methods based on bronchoscopic injection.

Dye injection is one of the first solutions implemented in the field of lung nodule localization, which was later applied to minimally invasive thoracic surgery [12]. The dyes, like blue methylene, have a short period of persistence on the lung surface, requiring lung resection to be performed a few hours after the marking procedure.

Nomori et al. [7] reported their experience with lipiodol and colored collagen for fluoroscopy-assisted resection. This technique requires the use of fluoroscopy in the operating room, which exposes the patient and the operating room staff to ionizing radiation. The authors reported that they performed the resection between 1 and 3 days after the marking procedure, suggesting a long-lasting characteristic of these markers.

Intraoperative ultrasound has been reported since the end of the previous century [13] to identify lung nodules intraoperatively. The procedure is usually followed by injection of dye. This technique is affected by an elevated rate of identification failure. One of the cases presented in the experience of Shennib et al. shows a distance of up to 2 cm between the marking and the actual position of lung nodules; this discrepancy is not acceptable in the era of sublobar resections. Moreover, as with other ultrasound procedures, this kind of marking is strictly operator-dependent. In the context of RATS with pericentimetric incisions, an ultrasound probe could be difficult to manage without extending the utility incision.

Abbas et al. [11] described their experience with ICG injection using electromagnetically guided bronchoscopy navigation. Their approach involves the use of software and equipment that is not available in all centers due to its cost, although it is a fully endoscopic solution that does not require injection into the lung parenchyma, thereby preventing the development of pneumothorax and pulmonary hematoma. Nevertheless, based on the images provided in their article, marking performed with their method appears to be more diffuse than our technique, thus reducing the accuracy of localization. This method seems to be very useful for delimiting the extension of lung parenchyma resection in segmentectomies or subsegmental resection, but less appropriate for small nodule identification. Furthermore, the resections were performed immediately after marking, providing no information about fluorescence or methylene blue persistence.

At the end of 1999, an Italian pilot study regarding 99mTc-labelled human serum albumin microsphere radionuclide use in pulmonary nodule localization was conducted [9]. Compared with our case series, it can be noted that the depth of the nodule from the visceral pleura is greater in both of our groups, especially in the Tech + ICG group. In contrast, their case series used a lower dose of radioactivity (5–10 MBq vs. 20–25 MBq). Their case history was referred to VATS resections, but the obtained results could also be applicable to robotic resection, demonstrating the reliability and performance of technetium-based marking.

In 2018, Nagai et al. published their retrospective case history about ICG marking of lung nodules [10]. The most relevant result, in our opinion, is the mean lung nodule depth of 9 mm (which is significantly less than our results), remarking on the strength of the addition of Technetium.

Theoretically, every injecting approach carries a risk of pneumothorax since it involves a transcutaneous puncture of the lung parenchyma. There is, however, a lower risk of bleeding or pulmonary hematomas compared to the placement of coils or metallic devices; metallic devices also have the risk of dislodgement in pleural space and migration [8]. A paper by Ichinose et al. [8] reported that 15.1% of complications were a result of hook wire devices, ranging from pulmonary hematoma to systemic air embolism; pneumothorax was detected by CT scan in 49% of the patients. This ratio of complications seems to be greater than other marking methods. In another case series, coil localization was found to be less frequently associated with dislocation and resulted in fewer complications than hook wire localization (25% vs. 54%) [14]. In this regard, it should also be noted that the coil positioning technique is more complex than that for injecting dyes or positioning hook wires; furthermore, the tail of the coil must remain visible on the visceral pleura [15].

Recently, Han et al. [16] presented a retrospective comparison of lung GGO localization using CT-guided hook wire positioning and CT-guided ICG injection. The resection was performed via VATS approach 3 h after the marking procedure. The ICG group demonstrated a lower respiratory discomfort immediately after the marking procedure, shorter time of localization, and fewer complications (lung hemorrhage, pneumothorax).

In conclusion, ICG and Technetium injection appear to be safer compared to hook wire or coil positioning techniques; in our experience, we report a total of seven marking procedure-related complications out of 83 patients (8.43%), all of which were managed conservatively.

The 99^m^Tc-MAA provides excellent depth penetration, allowing the surgeon to locate nodules that are not visible on the pleural surface, directly or indirectly, using the gamma probe. Applying pressure with the gamma camera to compress the lung parenchyma can also provide information about the depth of the nodule. Conversely, ICG provides an immediate visualization on the lung surface via NIR fluorescence (Figure 1), but it does not provide information about the nodule depth, and consequently, on the depth of the lung resection to be performed. If the marking procedure fails, Indocyanine Green is completely useless. The most common scenario, in the case of ICG marking failure, is leakage into the pleural cavity with the resulting indiscriminate marking of all structures (Figure 2). In these cases, it may be advisable to attempt to locate the nodule using an instrument of appropriate length (such as a VATS swab holder). Alternatively, a finger-guided localization could be attempted, enlarging one surgical access if necessary.

Our analysis of the variables suggests that while both methods are safe, the hybrid technique may offer a higher success rate in cases of deeper nodules, although differences are not statistically significant. Furthermore, the hybrid method did not show a significant increase in procedural complications compared to the Tech group, suggesting that the addition of ICG is a safe enhancement to the previous protocol.

We believe that the strengths of our technique lie in its ease of use (a single injection for both tracers), its precision, due to the procedure being performed under CT guidance by experienced operators, and the relatively low cost of the products used, which could potentially be available in all centers. The amount of radioactivity administered is minimal and has no significant impact on the patient or the operating room staff. The number of pneumothorax cases observed is acceptable given the total number of procedures, and furthermore, none of these required any intervention. We emphasize that the patient should undergo lung resection the day after the procedure; therefore, we consider the occurrence of this complication to be negligible unless it is of a very severe nature (massive pneumothorax). Our experience relates entirely to RATS resections, but the same technique can also be applied to VATS resections using an appropriate camera.

Finally, we would like to emphasize that close collaboration and communication among surgeons, radiologists, and nuclear physicians are essential to the success of the procedure.

In our case history, we found a lower conversion in the Tech + ICG group compared to the Tech-only group (6.55% vs. 13.63%), with all conversions being attributed to failure in nodule identification, although this difference was not supported by formal statistical significance due to the small sample size. We also performed a subset analysis comparing the conversion rate between the Tech and Tech + ICG methods based on the distance from the visceral pleura. Various thresholds were selected (15 mm, 20 mm, and 25 mm distance of the nodule from the visceral pleura), but statistical significance was not achieved in any case, even though the conversion rates were lower in the Tech + ICG group. The results of this analysis are reported in Table 2.

We are aware that the ability to feasibly perform nodule identification is influenced by many variables—such as lung quality (fibrosis, emphysema), the degree of insufflation, and the characteristics of the nodule (solid vs. subsolid)—and not merely by the distance from the visceral pleura.

From a histological point of view, both groups included a heterogeneous spectrum of lesions, ranging from benign nodules to invasive malignancies. This reflects real-world clinical practice, where preoperative diagnosis is often uncertain and surgical resection serves both diagnostic and therapeutic purposes. The high proportion of subsolid nodules highlights the importance of precise localization techniques, as these lesions are frequently early-stage adenocarcinomas or their precursors [6].

### Study Limitations

Despite these stimulating findings, several limitations must be acknowledged. First, the retrospective design introduces potential selection bias. Further studies, randomization, and larger surgical cohorts are needed; in fact, there is a lack of prospective studies that directly compare the performance of different marking systems. In addition, the uneven distribution of the patients between the two groups contributed to limiting the significance of the results.

## 5. Conclusions

The combination of Tech + ICG for pulmonary nodule localization seems to be an effective and reliable technique, despite all the statistically significant limitations. Compared to the use of 99^m^Tc-MAA alone, it seems that the Tech + ICG method facilitates easier identification of small or deep nodules during RATS, potentially leading to the improvement of surgical outcomes. We suggest the adoption of this dual-modality localization as a valid solution for pulmonary nodule identification; in fact, the hybrid approach is the one we have chosen for our clinical practice. The provided observations should be considered preliminary, as the results obtained are not statistically significant and the findings are based only on our clinical experience. We encourage other authors to share their experience in lung nodule marking.

## Figures and Tables

**Figure 1:**
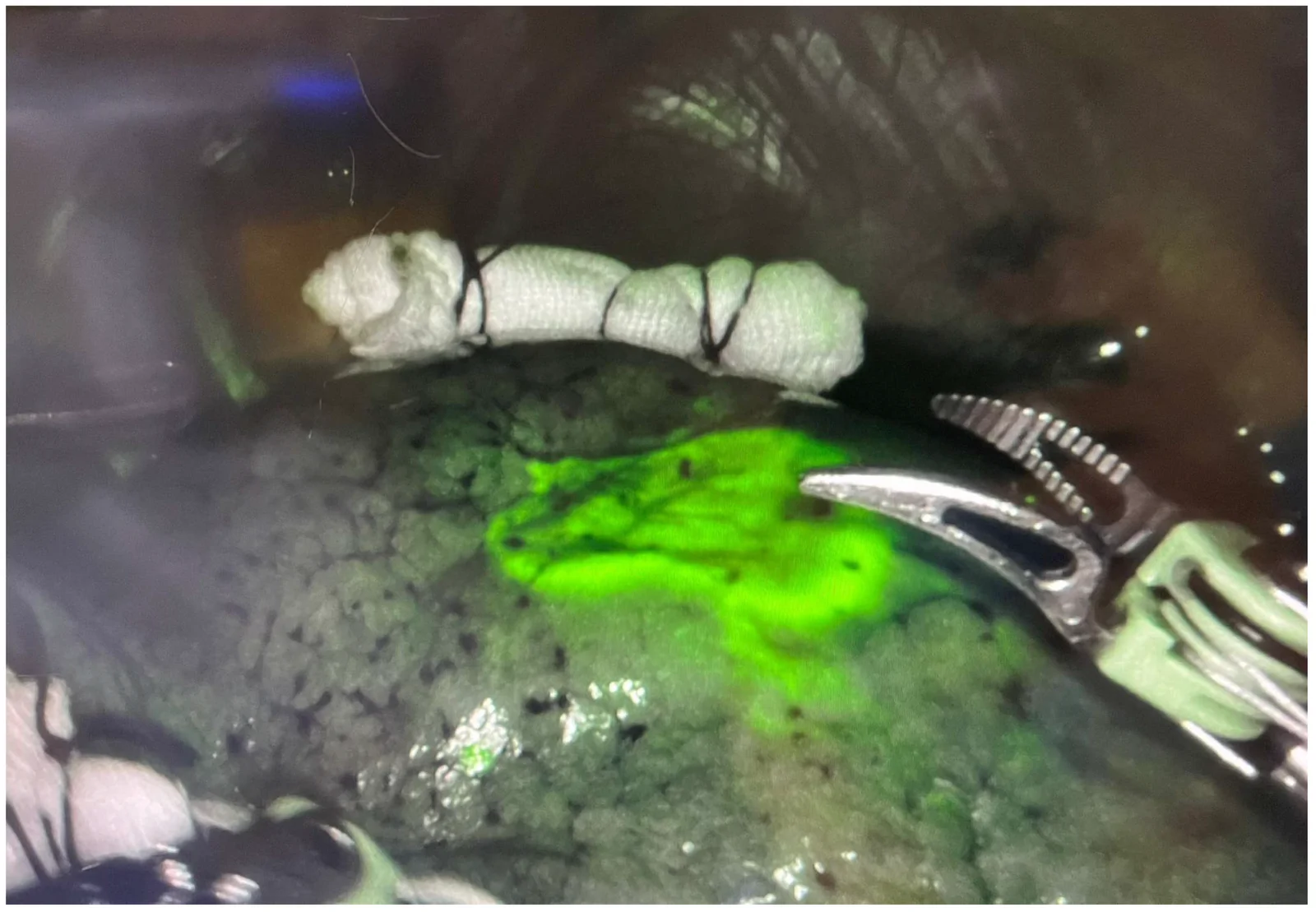
The dye is localized to the visceral pleura and is clearly distinct from the rest of the parenchyma (example of successful marking).

**Figure 2:**
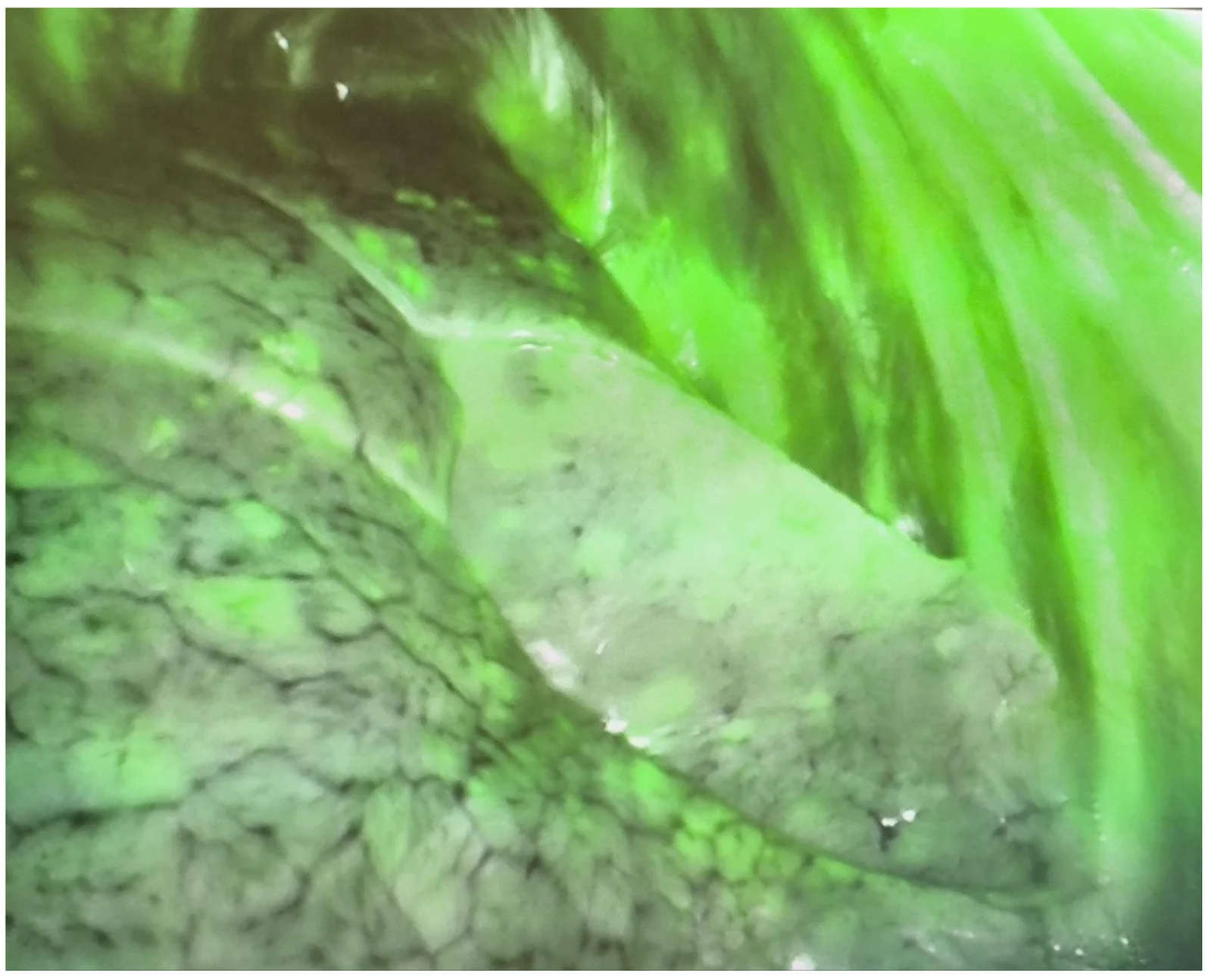
Failure of ICG marking. The dye was injected into the pleural cavity, uniformly staining the parietal and visceral pleura.

**Table 1:** Summary table for patients’ demographic information, nodule characteristics, procedure-related complications, histopathological findings, and length of stay.

	Tech Group (*n* = 22)	Tech + ICG Group (*n* = 61)
**Males (%)**	11 (50)	27 (44.26)
**Median age (IQR)**	50.5 (28.25)	44 (31)
**Median BMI (IQR)**	28.7 kg/m^2^ (IQR 7.2)	27 kg/m^2^ (IQR 8.9)
**Median nodule diameter (IQR)**	21.5 mm (16)	18 mm (14)
**Mean SUV max (SD)**	5.54 (±3.22)	5.69 (±3.23)
**Median distance of the nodule from visceral pleura (IQR)**	18 mm (21)	24 mm (15)
**Subsolid nodules (%)**	18 (81.82)	46 (75.4)
pure GGO	5	19
CTR < 0.5	7	17
CTR > 0.5	6	10
**Marking procedure-related complications (%)**	4 (18.18)	3 (4.91)
**Cases converted (%)**	3 (13.63)	4 (6.55)
**Mean operative time (SD)**	111.6 min (SD ± 45.6)	105.6 min (SD ± 36.8)
**Histology**		
Lung adenocarcinoma	13	12
Squamous cell carcinoma	1	7
Minimally invasive lung adenocarcinoma	2	9
Lung adenocarcinoma in situ	2	12
Typical carcinoid	-	1
Atypical adenomatous hyperplasia	-	7
Metastases from another tumor	3	7
Benign nodules	1	6
**Length of stay (SD)**	6 days (±2.8)	5.5 days (±2.9)

**Table 2:** Subset analysis of conversion rate based on the distance of the nodule from the visceral pleura.

Distance from Visceral Pleura	Conversion Rate in Tech Group	Conversion Rate in Tech + ICG Group	*p*-Value
<15 mm	12.5% (1/8)	0% (0/9)	0.471
≥15 mm	14.3% (2/14)	7.7% (4/52)	0.6
<20 mm	7.7% (1/13)	0% (0/20)	0.394
≥20 mm	22.2% (2/9)	9.8% (4/41)	0.293
<25 mm	6.7% (1/15)	0% (0/31)	0.326
≥25 mm	28.6% (2/7)	13.3% (4/30)	0.316

## Data Availability

The raw data supporting the conclusions of this article will be made available by the authors upon request.
